# Lots of Digital Files? How Digital Hoarding Is Related to the Academic Performance of University Students

**DOI:** 10.3390/ijerph22081186

**Published:** 2025-07-29

**Authors:** Natalia Bravo-Adasme, Alejandro Cataldo, Hedy Acosta-Antognoni, Elizabeth Grandón, Nicolás Bravo, Margarita Valdés

**Affiliations:** 1 Departamento de Sistemas de Información, Facultad de Ciencias Empresariales, Universidad del Bío-Bío, Concepción 4081112, Chile; natalia.bravo2102@alumnos.ubiobio.cl (N.B.-A.); egrandon@ubiobio.cl (E.G.); nbravo18@alumnos.utalca.cl (N.B.); mavaldes18@alumnos.utalca.cl (M.V.); 2 Escuela de Ingeniería Informática Empresarial, Facultad de Economía y Negocios, Universidad de Talca, Talca 3460000, Chile; 3 Faculty of Psychology, Universidad de Talca, Av. Lircay S/N., Talca 3460000, Chile; hacosta@utalca.cl

**Keywords:** digital hoarding, academic performance, academic burnout, academic engagement, mental health, higher education

## Abstract

Digital hoarding (DH) is an emerging behavior with potential implications for psychological well-being and daily functioning. While traditionally associated with physical hoarding disorder, DH presents unique challenges in digital environments, particularly among university students increasingly immersed in technology. This study examines the relationship between DH and academic performance, proposing a theoretical model in which academic engagement and academic burnout act as mediating mechanisms. Drawing on the Job Demands–Resources Theory, we provide evidence that DH contributes to a health impairment process that negatively affects student outcomes. Our findings reveal DH as a novel predictor of academic burnout, highlighting its detrimental impact on academic performance. These results carry significant theoretical and practical implications, offering new insights into the role of technology-related anxiety disorders in educational settings. From a practical perspective, our study underscores the need for higher education institutions to implement targeted interventions focused on emotional regulation and learning strategies to mitigate the negative effects of DH. Despite limitations related to sample specificity and cross-sectional data, this research opens avenues for future longitudinal studies and interventions aimed at addressing DH in both academic and professional contexts. By linking digital behaviors to mental health and performance, this work aligns with public health interests in understanding technology’s impact on youth well-being.

## 1. Introduction

In 2015, van Bennekom and colleagues reported the case of a 47-year-old man who was capturing and storing 1000 photographs daily on his computer [1]. More recently, FBI officials faced significant challenges analyzing the files of Sam Bankman-Fried, convicted of cryptocurrency fraud, due to the vast amount of data stored on his computer. Both cases illustrate behaviors that could be classified as “Digital Hoarding” [2], a phenomenon characterized by an uncontrolled need to store digital objects—such as photographs, emails, messages, web pages, and other binary files—and a marked difficulty in deleting them, ultimately affecting individuals’ daily functioning [3].

Digital hoarding shares conceptual similarities with a physical hoarding disorder [4,5], a recognized condition in which individuals struggle to discard possessions, which may be related to excessive accumulation.

In both cases, affected individuals experience distress when attempting to dispose of items, whether physical or digital. While digital hoarding may be considered a behavioral subtype of traditional hoarding, researchers have only recently begun to explore its nature and implications.

Emerging evidence suggests that digital hoarding is becoming an increasingly prevalent issue [6,7,8,9], with potentially serious consequences for individuals, organizations, and society at large. Although comprehensive studies remain limited, preliminary findings are concerning. A survey conducted by Western Digital revealed that, among 2000 Americans, 41% of respondents actively avoid deleting images and videos from their digital devices [10], while nearly a quarter (24%) admitted they would feel embarrassed if someone accessed the contents of their devices.

University students may be particularly vulnerable to digital hoarding. Since the onset of the pandemic, there has been a notable increase in technology use, a factor known to negatively affect college students’ well-being [11] and contribute to the development of mental health conditions [12]. This heightened exposure and near-ubiquity of digital environments may exacerbate the prevalence of digital hoarding among students. Given that digital hoarding is a relatively newly described behavior pattern, little is known about its impact on everyday functioning—particularly in academic contexts.

Prior research has suggested that digital hoarding might impair workers’ productivity [6,13,14]; however, these studies do not provide a clear explanation of the psychological mechanisms underlying this negative association. By extension, it is plausible that students affected by digital hoarding could also experience declines in academic performance. Thus, this study is guided by two research questions: Does digital hoarding affect the academic performance of university students? And what are the underlying psychological mechanisms through which digital hoarding might be related to such effects?

To address these questions, we propose an explanatory model that examines the relationship between digital hoarding and academic performance. Specifically, we analyze whether this relationship is mediated by academic engagement and academic burnout, using structural equation modeling in a sample of university students.

The remainder of this article is divided into six sections. The second section summarizes previous work. The third section describes the methodologies used. The fourth section presents the results. The fifth section discusses the results obtained. Finally, the main conclusions of this research are summarized.

## 2. Theoretical Framework and Hypothesis Development

### 2.1. Digital Hoarding

The concept of digital hoarding initially emerged in various blogs and websites. Users revealed their behaviors with their digital files, indicating they had thousands of unread emails, accumulated hundreds of messages on social platforms such as X or Facebook, or had thousands of photos stored on their cell phones, and expressing the difficulties involved in dealing with these on a daily basis [15,16]. However, it was not until 2015 that the concept of digital hoarding was first presented in scientific research [1]. It was established by the authors as a subtype of hoarding disorder and was defined as “the accumulation of digital files to the point of losing perspective, which eventually results in stress and disorganization” [1].

This definition emerged based on a 47-year-old male patient who kept digital photographs. He took up to 1000 landscape images daily and began to have difficulty deleting some of these photographs even though they were similar. The difficulty in discarding these images was mainly because he considered thy would bring back memories for him; he had an attachment to these photographs and was convinced that they would be useful to him in the future. Following this definition, some authors have begun to study digital hoarding and propose new definitions. Among these, it is possible to identify some common concepts, such as constant acquisition and the difficulty of discarding digital files as fundamental aspects [7,9,14,17].

As a relatively recent phenomenon, the causes that influence the development of this behavior have not been clearly identified. However, several authors mention that the increase in the use of several types of digital platforms and technological improvements in storage repositories have facilitated the accumulation of large amounts of digital content. The constant use of digital platforms and social networks has provided people with various options and facilities to develop digital content, which has led to the increase in such files and a greater number of options for sharing them [14]. Also, the technological development of storage repositories has enabled an increase in document storage capacity and a decrease in price to access more gigabytes of storage; this provides individuals with extensive options to accumulate digital files [4,7,14].

Additionally, the study conducted by Ref. [18] has established that there are four dimensions/motivations that can lead or drive digital hoarding: anxiety, compliance, disengagement, and collection. Anxiety-driven digital hoarding implies that anxiety drives individuals to store large amounts of digital files. This is mainly because individuals believe that they can use this information in the future and perceive some value from these documents. Disengagement-driven digital hoarding arises when individuals lack control over digital files, which begin to accumulate unnoticed. Collection-driven digital hoarding manifests itself in individuals making a conscious decision to store and not delete files.

Also, the literature has identified five barriers to deleting digital data [13]. The first barrier is keeping data for the future or just in case—for example, individuals do not delete emails or other files because they perceive that they may be useful in the future. The second barrier is keeping data as evidence; this barrier tends to arise more in work contexts, as individuals indicate that they primarily keep emails as evidence of work conducted and tasks sent or received. The third barrier is laziness, as deleting digital files is a time-consuming task, and individuals prefer to prioritize other tasks. The fourth barrier is emotional attachment to data, which is related to the sentimental value that people give to some files—mainly photographs, e-mails from friends or family, or music files with personal relevance. The final barrier is called “not my server—not my problem,” arising because of the increase in storage capacity, which reduces the pressure to delete files to free up space.

The authors state that digital hoarding can affect individuals in their personal and professional lives, as well as organizations. Regarding the effects on the individual, studies have identified that digital hoarding can affect the performance of daily tasks, disrupt sleep patterns, and decrease psychological well-being by creating nervousness, distress, frustration, stress, or anxiety [1,7,13,18]. Additionally, studies have identified a link between physical hoarding and digital hoarding, as some individuals who exhibit levels of physical hoarding have exhibited a greater tendency to hoard digitally [4,5]. In terms of the effects on organizations, digital hoarding has an effect on worker productivity, higher monetary cost in storage, and various cybersecurity risks. Storing many files in a company exposes it to possible malicious use of the stored material, both from within the organization and from external attacks [13,14,18,19,20].

### 2.2. Academic Engagement and Academic Burnout

The topics of academic well-being and academic distress have been acquiring great relevance because they are able to explain processes of (de)motivation and academic performance. Academic engagement has been defined as a construct with behavioral, cognitive and effective dimensions which refers to a positive, fulfilling, study-related state of mind characterized by vigor, dedication, and absorption [21]. The dimension of vigor refers to high levels of energy and mental resilience while studying, the willingness to invest effort in studying, and persistence even when encountering difficulties. The second dimension, dedication, refers to being strongly involved in one’s studies and experiencing a sense of significance, enthusiasm, inspiration, pride, and challenge. Absorption, the third dimension, refers to being fully concentrated and happily engrossed in what one is studying, where time passes quickly, and one finds it difficult to detach from studying [22].

In terms of well-being, academic engagement is seen as a key indicator in the prediction of positive educational outcomes and adaptive behaviors, where the most “engaged” students demonstrate more self-efficacy beliefs, positive coping strategies, effort, and perseverance; they are more enthusiastic about doing their homework and projects and are more motivated to learn and achieve more success in their education; and they experience lower levels of tension, stress, and anxiety [23,24]. Thus, Ref. [25] proposes them as an alternative for understanding the problems of performance, motivation, and academic dropout.

Ref. [26] explains that the interest in studying academic engagement is because it is a variable of vital importance for adolescents due to its relationship with academic adaptation and its protective role against risk behaviors.

Academic engagement has been studied as a motivational process over the last 25 years, with research mainly seeking to determine who the most engaged students are, why they are engaged, and what outcomes and consequences are produced by their high engagement [27,28]. Ref. [29] highlighted the importance of engagement in students, emphasizing that it was a relevant antecedent in learning activities. For example, Ref. [30] related academic engagement with positive emotions and academic performance in Chilean students. In this sense, assessing academic engagement is crucial because it is related to the retention and persistence of students during their studies [31]. Moreover, the assessment of engagement in academic contexts is relevant for the development of healthy educational contexts that support not only student outcomes (i.e., learning and performance) but also their optimal states of well-being [30]. According to the last study conducted by Ref. [30], academic engagement is positively associated with the dimensions of emotional and academic intelligence, and students who have higher levels of self-esteem are more committed and participative in their academic activities. In addition, Ref. [32] states that another reason for the growing interest in academic engagement is its potential applicability for interventions, as it can focus on interventions from a social or academic perspective, creating opportunities at school or in the classroom for participation, interpersonal relationships, and intellectual endeavors, and it can be a protective factor against dropping out of school [23].

Regarding academic distress, one of the causal constructs with vast empirical evidence is burnout [33]. In this way, according to Job Demands–Resources Theory [34,35] it is proposed that when an individual has insufficient resources to cope with the demands, academic or job burnout arises [36]. Burnout is considered a negative psychological syndrome in response to chronic stress related to the demands of the environment—in the case of students, their academic demands. Academic burnout regarding Ref. [37] involves negative attitude and behavior caused by learning pressure or a lack interest in learning. This syndrome has an impact on students’ motivation, satisfaction, learning, and outcome processes [38].

Ref. [39] reports that burnout among students showed a high prevalence of risk of mental disorders during adolescence. This chronic stress response is linked to the academic role, activity, and context [21] because academic life puts high pressure on students to perform successfully [40]. In addition, scientific evidence points to it being a good predictor of depression, suicidal intention, low academic performance [33], obsessive–compulsive disorders, phobic anxiety, or paranoid ideation.

Academic burnout, according to Ref. [38], is important for several reasons: (1) it is key to understanding a wide range of students’ negative behaviors during their studies; (2) it can influence their relationships, present and future, with their institution and with their fellow students, teachers, and others; and (3) it can affect higher education institutions through the academic dropout and failure rate, being a relevant aspect to evaluate the effectiveness of an institution. In this sense, Ref. [33] conducted a systematic review of academic burnout in nursing students, showing that there is a negative relationship between burnout, self-concept, and academic engagement.

Ref. [21] proposed three dimensions for understanding academic burnout: exhaustion, cynicism, and inefficacy. Burnout is characterized by a feeling of being exhausted, of being unable to cope with tasks as a student. Cynicism refers to a cynical or distancing attitude towards the meaning and usefulness of the studies being carried out. Finally, inefficacy is a feeling of academic incompetence as a student. Ref. [41] conducted a systematic review regarding the prevalence of burnout in university students, examining 20 studies (5% from North America, 25% from Asia, 45% from Latin America, and 25% from Europe) and estimating that 55.4% of students have burnout, 31.6% have cynicism, and 30.9% have inefficacy. They concluded that the university student population presents moderate levels of burnout.

### 2.3. Job Demands–Resources Theory

The Job Demands–Resources (JDR) model is one of the most widely recognized theoretical frameworks in occupational health psychology and organizational behavior. Originally proposed by Ref [42], the JDR model posits that job characteristics can be categorized into two broad dimensions: job demands those physical, psychological, social, or organizational aspects of work that require sustained effort and are associated with physiological and psychological costs and job resources which refer to those elements that reduce job demands and their associated costs, foster personal growth, and enhance work engagement and performance. According to the model, high job demands may lead to strain and burnout (the health impairment process), whereas sufficient job resources can trigger motivational processes that result in increased engagement and positive outcomes [42,43].

The JDR model is particularly suitable for analyzing phenomena such as digital hoarding (DH) among students due to its ability to capture the complex interactions between demands and resources in digital academic environments. This theoretical framework focuses on how job characteristics (in this case, academic demands and available resources) influence individuals’ psychological well-being and performance [43]. In the context of digital hoarding, demands may include information overload, the pressure to maintain multiple digital platforms, or the need to manage large volumes of files and data. On the other hand, resources could include technological skills, institutional support, or organizational tools that facilitate the efficient management of digital information.

Compared to competing theories, such as Karasek’s job demands–control model [44] or the HERO model (Healthy and Resilient Organizations) [45], the JDR model offers a significant advantage by considering both demands and resources simultaneously, allowing for a more comprehensive understanding of the effects of digital hoarding on students. Karasek’s model, for example, primarily emphasizes the relationship between job demands and control, but does not provide a complete framework for understanding how specific resources can mitigate the negative effects of these demands [44,46]. Meanwhile, the HERO model, although valuable for understanding resilient organizational practices, lacks the necessary precision to explain specific dynamics related to digital hoarding and its impacts on variables such as academic engagement or burnout [45]. Additionally, the JDR model has proven highly relevant in educational contexts, where demands and resources are closely linked to academic performance and student well-being. Previous studies have successfully applied the JDR model in higher education settings [34].

### 2.4. Theoretical Gap and Hypothesis Proposal

Research on digital hoarding is recent and limited [4,13,20,47], and it has mainly focused on measuring individuals’ level of digital hoarding of certain digital items (documents, emails, images, or videos) in personal or work environments through instruments originally used to measure physical hoarding or by adapting these to measure digital hoarding [5,6,7,13,14]. Also, very little research has examined the digital hoarding construct as a predictor. Among the main contributions are those made by Ref. [9] who established that digital hoarding is related to the stress perceived by individuals and by Ref. [7], who provide evidence confirming the association between digital hoarding and anxiety.

In the case of the student population, research is scarcer and has focused on analyzing the relationship between digital hoarding and hoarding disorder symptoms [4] and on identifying the subdimensions of digital photo hoarding [47]. Additionally, Ref. [17] analyzed the relationship between digital hoarding behaviors, computer self-efficacy, and computer anxiety in students. It has not been possible to identify studies that analyze digital hoarding in students and its effect on academic engagement, academic burnout, and academic performance. This population is interesting to study, as it could be expected to demonstrate different behavior than the worker population because university students are a generation of digital natives, i.e., they were born surrounded by various technologies and technological developments and the use of digital technologies are a fundamental part of their daily activities [48]. Therefore, their adaptive behavior in the face of these developments could be expected to be different from those who have had to learn to use them.

#### 2.4.1. Academic Engagement and Academic Performance

From the Job Demands–Resources Theory [35] the motivational process explains that the optimal resources to cope with the demands allow engagement and positive performance. In this line, academic engagement is a predictor of academic performance; this is because when students are engaged, they tend to make an effort and use all the resources available to achieve their goals, so they manage to learn better and reach better results [34]. Engaged students are characterized by showing greater energy, enthusiasm, dedication, and enjoyment in their academic activities and tasks, which is positively related to high academic performance [27].

The studies that analyze this relationship have been conducted at different educational levels: in primary education, high school, and university populations [49], and most of them have established a positive relationship between academic engagement and academic performance. Moreover, authors who studied this relationship in higher education students suggest that academic engagement is one of the key variables that explain academic performance. Research shows that academic engagement is evidenced in students through higher levels of vigor and dedication in their academic activities, which may be related to higher academic performance [30]. This leads us to propose the first hypothesis of our explanatory model:

**H1.**  *Higher levels of academic engagement are related to higher levels of academic performance.*

#### 2.4.2. Academic Burnout and Academic Performance

According to Job Demands–Resources Theory, the health impartment process explains that reduced resources to cope with high demands can lead to the development of burnout [35]. For students, achieving adequate academic performance is a goal that determines their success. Entering a health impairment process, such as academic burnout, has a relationship with low academic performance. This means that students with high burnout, high cynicism, and low academic self-efficacy obtain low results in academic evaluations [33,50].

Studies have established that there are common factors when a student moves from elementary to high school that lead to academic burnout and can lead to low levels of academic achievement [51]. Research conducted on high school and university students found that academic burnout predicts and negatively affects academic performance [52]. The authors propose that academic burnout manifests in students through a perceived high academic load, lack of enthusiasm, absenteeism, and low engagement which results in lower academic performance [53]. This leads us to propose the second hypothesis of our explanatory model:

**H2.**  *Higher levels of academic burnout are related to lower levels of academic performance.*

#### 2.4.3. Digital Hoarding and Academic Engagement

The current literature on digital hoarding is new, and it has not been possible to identify studies that relate digital hoarding and academic engagement. However, one study has analyzed the relationship between physical hoarding and work engagement. This study established that individuals with hoarding disorder tend to show personal difficulties or attention problems [54] which may be associated with lower work engagement. Although the authors hypothesized that there would be a negative relationship between hoarding and work engagement, the results determined that there is no significant relationship [55].

Regarding the relationship between digital hoarding and academic engagement, a student might be expected to possess an accumulation of several digital items (e.g., papers, lecture slides, reading material, audio recordings of lectures, or photographs of their classes). In many cases, these files will be disorganized and even duplicated or triplicated in different repositories (e.g., computer, phone, tablet, and/or the cloud). This makes the search and identification of a necessary file—for example, a document needed to study—become tedious and involve time that the student considers valuable, leading them to consider the experience as negative, which can be reflected in lower academic engagement. Based on the above, we propose the third hypothesis of this research:

**H3.**  *Higher levels of digital hoarding are related to lower levels of academic engagement.*

#### 2.4.4. Digital Hoarding and Academic Burnout

Studies that have sought to identify similarities between physical hoarding and digital hoarding have used questionnaires that measure obsessive–compulsive disorders [4,5]. This is because “Hoarding” is one of the subscales of the questionnaire, so individuals with OCD would be expected to have characteristics in common with those of a physical hoarder. A study conducted on higher education students determined that there is no significant relationship between the Hoarding subscale and the academic burnout subscale [56].

Students are constantly receiving digital files, which in most cases are stored in a disorganized manner. This situation will not generate problems for the student; however, inconveniences will arise when they need to quickly find a file for the delivery of a final assignment in a course or to prepare for an exam. At that point, the student will realize how many files they have and that even the specific document they need may exist in several versions or be stored with the same name. These situations, which in some cases may be urgent, can generate stress and anxiety and increase burnout levels in the student. Based on the above, we establish the fourth research hypothesis:

**H4.**  *Higher levels of digital hoarding are related to higher levels of academic burnout.*

#### 2.4.5. Digital Hoarding and Academic Performance

Research analyzing the relationship between digital hoarding and academic performance is scarce. However, there are some studies that have managed to identify how digital hoarding influences individual and work performance. In the case of individual performance, hoarding digital items involves significant time dedication, which decreases performance in other task areas [1]. Additionally, studies in work contexts established that individuals who accumulated many digital files perceived a decrease in their productivity [13]. In many cases, individuals tended to save files as future evidence of the work performed; permanently saving files implied the use of time that could be used in activities directly related to their work [6].

Students tend to store different files with similar names, labels that do not allow for identifying what the document is about, or duplicate files on the same or different devices. Trying to find a specific document will require the student to use more time that could be used for other academic activities, affecting their productivity and, in some cases, their academic performance. Based on the above, we propose the fifth hypothesis of the study:

**H5.**  *Higher levels of digital hoarding directly and negatively related to academic performance.*

**Figure 1:**
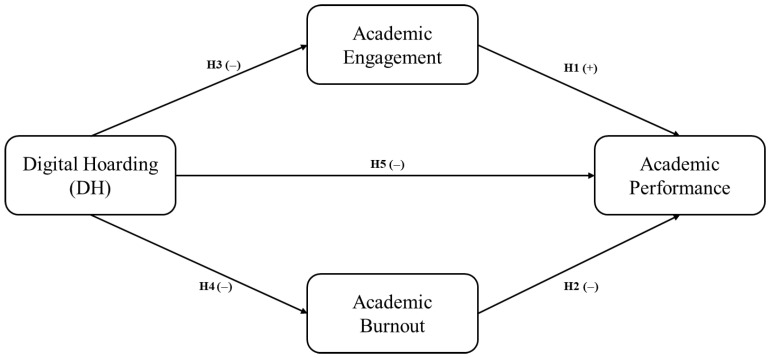
Theoretical model of this research.

Figure 1 shows a schematic diagram of the theoretical model studied.

## 3. Materials and Methods

Data collection was carried out through the application of a self-administered questionnaire. The studied population specifically corresponds to full-time university students from a public higher education institution in Chile. Participants were recruited in common areas and spaces across the university campus. The eligibility criteria established were being a university student of legal age, having grades recorded during the semester in which the study is being conducted, and using technological devices such as computers, tablets, smartphones, or similar tools for academic activities. These criteria were applied to ensure the feasibility and consistency of participation throughout the study. The instrument was constructed with questions adapted and validated in previous research (Table 1).

The questions were translated and adapted with special care to maintain their original meaning. The translation and adaptation of the items used in this study were carried out through a rigorous process to ensure their accuracy, linguistic appropriateness, and cultural relevance. The items from Refs. [6,28], originally in English, were translated into Spanish through a procedure that began with two independent translations conducted by bilingual researchers (English–Spanish). Based on these versions, a preliminary translation was developed and subsequently reviewed and validated by two additional bilingual researchers with expertise in research methodology and the study’s thematic area. Throughout this process, a critical analysis was conducted to ensure semantic and cultural equivalence, assessing not only the literal accuracy of terms but also their contextual meaning and relevance within the Spanish-speaking context. Minor adjustments were made to the language to enhance clarity and contextual appropriateness, without altering the original conceptual intent of each item. On the other hand, the items derived from Ref. [30] were already available in Spanish and were therefore used without modifications, as their wording was deemed suitable for the context of the present study.

The survey has 10 items corresponding to the two dimensions of digital hoarding (difficulty in deleting and in accumulating), nine items pertaining to the three dimensions of academic engagement (vigor, dedication, and absorption), nine items corresponding to the three dimensions of academic burnout (study exhaustion, cynicism towards the meaning of study, and feeling of inadequacy as a student), and five items related to academic performance. Additionally, two security items were incorporated to ensure the consistency of the responses. The rest of the questionnaire consists of demographic questions such as gender, university affiliation, degree program, and current academic year, as well as questions about the digital devices and digital content the participant owns. Additionally, it includes items regarding the participants’ sleep patterns.

The digital hoarding questions were measured using a seven-point Likert scale (from “Strongly Disagree” to “Strongly Agree”), the academic burnout items were measured using a six-point Likert scale (from “Never” to “Always”), the academic engagement items were measured using a six-point Likert scale (from “Strongly Disagree” to “Strongly Agree”), and the academic performance items were measured using a five-point Likert scale (from “Strongly Disagree” to “Strongly Agree”).

A pre-test was administered to 12 undergraduate students. Based on feedback from the participants, modifications were made to four items for better understanding. All responses obtained in the pre-test period were not incorporated in the final sample analyzed. After the modifications made during the pre-test, information was collected through the face-to-face application of the survey in various institutions of higher education. Finally, a sample of 327 valid questionnaires remained to be analyzed.

For the analysis, we used a second-order structural equation model (PLS-SEM), which has advantages over other methods such as CB-SEM. PLS-SEM should be used when (a) a small population restricts the sample size, (b) distribution issues, such as lack of normality, are a concern, (c) the analysis is concerned with testing a theoretical framework from a prediction perspective, or (d) the structural model is complex [58,59].

The digital hoarding construct (DH) was modeled as a second-order reflexive-reflexive construct consisting of two first-order constructs, corresponding to the two dimensions of digital hoarding: Digital Hoarding Difficulty Deleting (DHD) and Digital Hoarding Accumulating (DHA). The construct of Burnout (BO) was also modeled as a second-order reflexive-reflexive construct and consisted of three dimensions: Cynicism Academic Burnout (CBO), Exhaustion Academic Burnout (EBO), and Inadequacy Academic Burnout (IBO). The construct of Academic Engagement (AE) was modeled as a second-order reflexive-reflexive construct and consisted of three dimensions: Vigor Academic Engagement (VAE), Dedication Academic Engagement (DAE), and Absorption Academic Engagement (AAE). Academic performance was modeled in two ways: a first-order construct composed of four items (PAP) and a single-item construct of the self-perceived score (OAP).

## 4. Results

A total of 333 surveys were received, but six were discarded due to validation issues. These were discarded during the data cleaning process because they did not answer one or both security questions included in the questionnaire to measure response consistency. A total of 215 (65.74%) respondents were women. The respondents were between 18 and 38 years old. Three respondents did not identify as man or woman. The median auto-perceived score in percentage was 70.0% in men, 73.3% in women, and 73.3% in non-man and non-woman. The minimal number of devices owned by students was one, and the maximum was fourteen. Table 2 shows the descriptive statistics of the respondents.

The surveys were conducted with students enrolled in higher education institutions. Data was collected in person through a self-administered questionnaire, using a convenience sampling method. Participation was voluntary, and no monetary or material compensation was provided to the respondents. Prior to completing the questionnaire, all participants were informed about the purpose of the study and signed an informed consent form to confirm their voluntary participation.

Three separate criteria suggest that the sample is large enough. First, according to the 10 times rule [60], the sample size exceeds the recommended minimum (30 cases). Second, the sample size also exceeds the criteria established to achieve a statistical power of 80% for detecting R^2^ values of at least 0.10 with a 5% of significance (103 cases) [59]. Third, the inverse square root method [61] utilizes the minimal path to calculate the minimal sample size, assuming a minimum path coefficient expected between 0.11 and 0.20, where 155 observations would be an acceptable minimal [59]. Later, our results confirmed a minimal sample of 303 participants (minimal significant path of 0.146). The sample size exceeds all these thresholds.

Two measures were taken to decrease common method bias risk in the questionnaire: different scales were used in the digital hoarding, engagement, burnout, and performance constructs, and questions were reversed in the items of both constructs. Furthermore, to check for common method bias, we ran Harman’s single-factor test. The results confirm that there is no problem with common method bias in the data since the total variance extracted by one factor is 23.7%, which is less than the recommended threshold of 50%.

Our analysis of the second-order model was based on a two-stage process, following the recommendations of Ref. [58]. In the first stage, we used the repeated indicator approach to obtain the latent variable scores for the lower order constructs (LOCs). Furthermore, we adjusted the outer model to achieve an acceptable level of reliability and validity. In the second stage, we used the latent variable scores as manifest variables in the second-order measurement model. Next, we present the results of the two stages.

### 4.1. First-Stage Analysis

We first examined all first-order reflexive constructs (auto-perceived academic performance, PAP) based on the factor loadings (where loads should be greater than 0.6–0.7 for exploratory studies), composite reliability, and average variance extracted (AVE) [59]. Two items were removed because the load was below the acceptable threshold, AP2 and AP4. Next, we adjusted the LOCs, i.e., the dimensions of digital hoarding, academic engagement, and burnout [58]. Based on factor loadings, reliability, and validity criteria, one item was removed from digital hoarding (DHD6). Similarly, two items were removed from academic engagement (AA1 and VAE3). One item was removed from academic burnout (IBO1). In total, six items out of 33 were removed from the entire model—18.2%, which is below the maximum acceptable limit of 20% to avoid problems in measuring the constructs. Supplementary Table S1 shows the estimates for all constructs of the first-stage model.

As suggested by Ref. [58], the assessment of HOC reliability and validity must be manually calculated. In reflective-reflective constructs, path coefficients correspond to loadings [59]. As the reader can see in Supplementary Table S1, the reliability and validity of digital hoarding, academic engagement, and burnout were over the threshold suggested, i.e., composite and Cronbach’s alpha over 0.7 and AVE over 0.5, thus confirming the reliability and validity of the outer model.

Discriminant validity was assessed using the heterotrait–monotrait ratio criterion (HTMT), finding a value lower than 0.90, which suggests a good discriminant validity. In the first-stage analysis, discriminant validity must be established between all first-order constructs, and it is not necessary to achieve discriminant validity between first-order and second-order constructs [58]. Supplementary Table S2 shows the HTMT for all first-order constructs, and it can be seen that all indicators are within the acceptable range.

### 4.2. Second-Stage Analysis

Next, we advanced to the second stage of the analysis. Firstly, the LOC scores served as manifest variables in the two second-order constructs (i.e., digital hoarding, academic engagement, and academic burnout) of the measurement model. Table 3 shows the validity, and reliability estimates for the second-order model. As can be seen, all constructs are within the recommended thresholds, showing that the second-order outer model meets the validity and reliability requirements.

After evaluating the outer model, we assessed the structural model by implementing a bootstrapping method (5000 subsamples, BCa bootstrap, two-tailed, 5% level of significance, factor weighting scheme). Table 4 presents the hypothesis assessment.

Table 4 shows that four hypotheses are confirmed. Digital hoarding is positively associated with academic burnout (H4). In turn, burnout is negatively related to academic performance measured through auto-perceived academic performance and auto-perceived score (H2a and H2b, respectively). Academic engagement is related only to auto-perceived academic performance (H1a), but not to auto-perceived score (H1b). However, if we adjust the significance level to 10%, academic engagement is positively associated with academic performance. Digital hoarding is not related to academic engagement.

Table 5 summarizes the coefficients of determination (R^2^) and Q^2^. R^2^ values indicate the explanatory power of the model’s endogenous variables. Q^2^ values (Stone-Geisser) indicate the model’s out-of-sample predictive power.

Interestingly, digital hoarding does not directly relate to academic performance on either measure. This shows that the mechanism by which digital hoarding affects students’ academic performance is academic burnout. A post hoc mediation analysis confirms this result (see Table 6). That is, academic burnout fully mediates the relationship between HD and academic performance. It is worth noting that digital hoarding is only associated with auto-perceived academic performance (PAP) and not the auto-perceived score (OAP).

**Figure 2:**
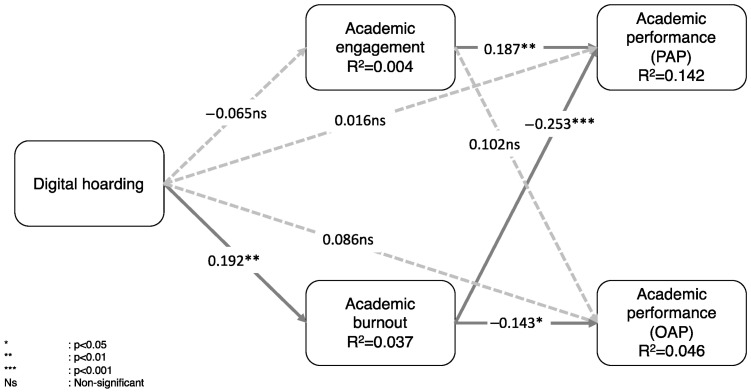
Final model with the hypothesis analysis.

Figure 2 shows that both academic engagement and academic burnout explain 14.2% of the variance of auto-perceived academic performance (PAP) and 4.6% of the variance of the auto-perceived score (OAP). The most influential construct of academic performance is burnout. The Q^2^ value indicates the model’s out-of-sample predictive power, with values of Q^2^ larger than zero indicating that the exogenous constructs have predictive relevance for the endogenous construct under consideration. The results show that the model has predictive relevance for all endogenous constructs.

## 5. Discussion

The main purpose of the present study was to explain the relationship of digital hoarding on academic performance in higher education students. Also, it aimed to analyze the relationship between DH and academic performance, mediated by academic engagement and academic burnout as an underlying psychological mechanism, in a sample of university students. Our results show that digital hoarding is not related to academic engagement, and academic engagement is not related to auto-perceived academic performance. Additionally, digital hoarding is positively related to academic burnout. In turn, academic burnout is negatively related to academic performance. Finally, the results show that digital hoarding does not have a direct relationship with academic performance measured through auto-perceived score (OAP).

Although no studies have been identified that analyze the relationship between digital hoarding and academic engagement, our results are consistent with studies that analyzed the relationship between hoarding disorder and work engagement. These studies determined that there is no significant relationship between the two constructs [55]. Our results could be explained, on the one hand, as a consequence of online classes. Many students access classes remotely, which implied that reports, presentations, and even tests had to be rendered digitally, increasing the storage of digital content. This situation may have led students to become used to the constant use and accumulation of digital files in their academic activities, and because of this there were no repercussions in their academic engagement. Another possible explanation is the age of the individuals analyzed: they are university students, mostly between 18 and 25 years old. This population is known in the literature as digital natives—people who were born surrounded by technological developments [62,63], so it would be expected that they can better cope with the consequences of ICT use, as would be the case of digital hoarding.

Regarding the positive relationship of academic engagement on academic performance measured through auto-perceived academic performance (PAP), this is consistent with previous literature that has studied this relationship [30,49]. Several authors indicate that academic engagement is one of the factors that can positively explain academic performance, suggesting that the more engaged students are, the easier it will be for them to achieve their academic goals [64]. Our results can be explained by the fact that a significant portion of the respondents were in their first years of university. In these initial stages, where students make the transition from the school stage to the university stage, a high level of involvement tends to be required to achieve the objectives; students would be expected to show greater engagement and enthusiasm for learning, which could be related to better academic.

Additionally, our results provide support that digital hoarding is positively related to academic burnout, and, in turn, academic burnout is negatively related to performance of higher education students. This is in part consistent with previous research that determined that DH could have negative effects on individuals, such as stress and anxiety [1,7,9]. However, there are also studies that have analyzed the hoarding scale in people with obsessive–compulsive disorders and academic burnout, determining that there is no significant relationship between the two [56]. The results could be explained by how students store large amounts of digital content related to their academic activities and much of it is not deleted because it may be necessary for their studies or used in the future, or simply because the students are too lazy to delete files. Therefore, these files are constantly being stored in a disorganized manner, often with similar names or attributes that do not allow for clear identification of the document. This lack of organization may contribute to increased stress or frustration for the student when urgently trying to locate an important file, potentially leading to higher levels of burnout.

Our findings show that academic burnout is negatively related to the academic performance of higher education students and agree with the current literature [30,33,53]. These results can be explained by the fact that higher education students are in constant academic demand, which increases further during the end-of-year periods. This can lead students to present high levels of emotional exhaustion, feelings of constant fatigue, or a lack of interest in fulfilling their academic commitments, or they may not feel that they have the necessary skills to fulfill these commitments. Another possible explanation is the students’ lack of sleep. Several participants reported a lack of balanced sleep due to their academic activities in the questionnaire, which may also explain the high levels of academic burnout and its negative effect on performance.

As noted in Table 5, some key constructs exhibit low coefficients of determination (R^2^). Two specific cases warrant further clarification: Academic Engagement (AE) and Academic Burnout (BO). For Academic Engagement, the minimal R^2^ value (0.004) aligns with the nonsignificant relationship between its antecedent, Digital Hoarding (DH) and AE (β = −0.065, *p* > 0.05). This suggests that DH does not meaningfully influence AE in our model. Conversely, Academic Burnout demonstrates a statistically significant relationship with DH (β = −0.192, *p* < 0.01), albeit with a low R^2^ (0.037). Burnout is inherently a multifaceted construct influenced by diverse factors, including academic pressure, social dynamics, emotional support, and individual traits like self-esteem or mental health. In such contexts, even a small R^2^ (e.g., 0.037) may hold practical relevance when considering the complexity of antecedent-outcome relationships. Similarly, academic performance statistics (R^2^ = 0.142 and 0.046) reflect the inherent challenges of predicting outcomes shaped by numerous interrelated variables, such as personal motivation, institutional resources, and external stressors. Low R^2^ values in such cases should not automatically negate the validity or utility of observed relationships.

While the explained variances of our model are limited (low R^2^), it is critical to emphasize the predictive relevance indicated by the Stone–Geisser Q^2^ criterion. Positive Q^2^ values across all four endogenous constructs suggest that the model retains predictive capability, even if it explains only a modest proportion of variance. This divergence between explanatory and predictive performance is particularly pertinent for constructs like Burnout and Academic Performance, which are influenced by myriad dynamics and often unobserved factors. Thus, the model’s predictive utility complements its theoretical insights.

Finally, the results show that DH is not directly related to the academic performance of higher education students. This is contrary to studies analyzing the relationship between DH and individual and work performance, which identify that people who hoard digital content use a lot of time to manage it, perform less well in other tasks, and have lower work productivity [1,6,13]. Because current university students have developed their learning processes surrounded by technology and digital content, they deal with its consequences in a good way and do not see their academic performance affected. Research on digital hoarding is in an emerging process of study and does not yet have a solid theoretical basis [7], so we recommend analyzing these constructs in greater detail to achieve more conclusive results.

Our findings offer some reflections for the ongoing development and contextual adaptation of the Job Demands–Resources (JDR) theory. Although the JDR has been widely applied in organizational and educational contexts to explain how job demands and resources influence well-being and performance through mechanisms such as exhaustion and engagement [42,43], our study reveals certain limitations in applying this framework to emerging phenomena such as digital hoarding among university students. Specifically, the absence of a significant relationship between digital hoarding and academic engagement challenges the assumption that high demands generally have negative effects on motivational outcomes. This suggests that in deeply digital environments, particularly among digital natives, certain demands do not necessarily interfere with engagement, possibly due to familiarity, adaptive coping strategies, or changes in how these demands are perceived because of the common use of digital tools.

## 6. Conclusions

As we mentioned in the introduction, digital hoarding is a behavior worthy of study due to its impact on the lives of those affected by it. We conducted a study to analyze the relationship between DH and academic performance. Additionally, our model proposes an undelaying psychological mechanism in which the relationship of DH on performance is mediated by academic engagement and academic burnout.

This study has theoretical and practical implications. From theoretical implications, firstly, our study following the Job Demand–Resources Theory provides evidence that the health impairment process [35] is relevant to the academic performance of university students. Secondly, our study proposes a new predictor of academic burnout, that is, DH. Finally, this study has theoretical and practical implications. Drawing from theoretical implications, firstly, our study, following the Job Demand–Resources Theory, provides evidence that the health impairment process [35] is relevant to the academic performance of university students. Secondly, our study proposes a new predictor of academic burnout, that is, DH. Finally, the study attracts attention to an emerging phenomenon within the university context by emphasizing the detrimental outcomes of academic burnout, particularly its impact on students’ academic performance.

Looking at the practical implications, we can highlight that mental health issues among students pose a significant challenge for higher educational institutions. Research indicates that depressive–anxious disorders in these students can lead to substance abuse, suicidal tendencies, and risky and violent behaviors, as well as can increase the risk of future mental disorders [65]. The transition back to face-to-face learning has amplified these risks, placing greater pressure on young individuals and straining university and college budgets. Unfortunately, the resources available to institutions for supporting students are limited. Consequently, the ability to more accurately diagnose the specific types of disorders experienced by students would facilitate a more efficient allocation of these resources. Therefore, the findings of this study are of great interest to higher education institutions, as they can help identify whether affected students are experiencing a novel anxiety disorder directly linked to the use of technology. In this way, universities could implement diagnosis and interventions focused on increasing personal (i.e., emotional regulation) and academic resources (learning strategies) as organizational plans to decrease the levels of health impairment processes in their students.

This study has some evident limitations. The primary limitation of the study lies in the specific and limited nature of the analyzed population. Since the group of participants was restricted to university students, this limits the possibility of generalizing the results to other contexts or population groups. Therefore, it would be premature to claim that the findings are applicable to other groups such as workers or employees in work environments. However, these results serve as a starting point for future research that should explore the validity of these findings in more diverse samples. A second limitation refers to the data that were cross-sectional, and causal effects cannot be provided. Nevertheless, our study raises several inquiries that should be addressed in future research. For instance, do workers with digital hoarding disorder experience any impairment in their performance or productivity? Furthermore, does digital hoarding once again associate with workers’ burnout, and does burnout, in turn, affect their performance?. Longitudinal studies could also be conducted to explain the causes and effects of this behavior, addressing virtuous or deteriorating spirals over time. Likewise, interventions could be implemented and their effectiveness tested in the context of university students.

## Figures and Tables

**Table 1:** Questions by construct and reference source.

Construct	Dimension	Number of Items	Reference
Digital Hoarding	Difficulty deleting	6	[6]
	Accumulating	4	
Academic engagement	Vigor	3	[30]
	Dedication	3	
	Absorption	3	
Academic Burnout	Exhaustion from studying	4	[28]
	Cynicism toward the meaningfulness of studying	3	
	Sense of inadequacy as a student	2	
Academic Performance	Auto-perceived academic performance	4	[57]
	Auto-perceived score	1	

**Table 2:** Descriptive statistics.

	Sex	N	Mean	Median	SD	Minimum	Maximum
Age	M	109	20.730	20	2.380	18	28
	F	215	20.800	20	2.740	18	38
	Another	3	19.670	19	2.080	18	22
Auto perceived Score in %	M	109	70.640	70.0	9.660	41.7	91.7
	F	213	72.330	73.3	10.910	16.7	96.7
	Another	3	70.000	73.3	10.410	58.3	78.3
Total Devices	M	109	2.620	2	0.790	1	5
	F	215	2.710	3	1.090	1	14
	Another	3	2.000	2	0.000	2	2

**Table 3:** Validity and reliability estimates for the second-order model.

	Loading	CA	CR	AVE	HTMT			
					PAP	BO	DH	AE
PAP		0.681	0.861	0.756				
R1	0.836							
R3	0.901							
BO		0.818	0.892	0.734	0.453			
CBO	0.894							
EBO	0.804							
IBO	0.870							
DH		0.732	0.869	0.770	0.091	0.228		
DHA	0.791							
DHD	0.956							
AE		0.828	0.896	0.742	0.386	0.559	0.066	
AAE	0.832							
DAE	0.926							
VAE	0.824							
OAP	1.000	1.000	1.000	1.000	0.452	0.193	0.055	0.176

CA: Cronbach’s Alpha; CR: Composite Reliability; AVE: Average variance extracted; HTMT: Heterotrait–monotrait ratio. AE: Academic engagement; BO: Burn-out; DH: Digital hoarding; PAP: Auto-perceived academic performance; OAP: Auto-perceived score. AAE: Absorption Academic Engagement; VAE: Vigor Academic Engagement; DAE: Dedication Academic Engagement; CBO: Cynicism Academic Burnout; EBO: Exhaustion Academic Burnout; IBO: Inadequacy Academic Burnout; DHA: Digital Hoarding Accumulating; DHD: Digital Hoarding Difficulty deleting.

**Table 4:** Hypothesis assessment of the structural model.

Hypothesis	Relationship	Path Coeff.	*p* Values	Conclusion
H1a	AE → PAP	0.187	0.001	Supported
H1b	AE → OAP	0.102	0.093	Not supported
H2a	BO → PAP	−0.253	0.000	Supported
H2b	BO → OAP	−0.143	0.037	Supported
H3	DH → AE	−0.065	0.308	Not supported
H4	DH → BO	0.192	0.001	Supported
H5a	DH → PAP	0.016	0.795	Not supported
H5b	DH → OAP	0.086	0.172	Not supported

AE: Academic engagement; BO: Burn-out; DH: Digital hoarding; PAP: Auto-perceived academic performance; OAP: Auto-perceived score.

**Table 5:** Explanatory (R^2^) and predictive (Q^2^) power of the model.

	R^2^	Adjust R^2^	Q^2^	Predictive Power (Q^2^ > 0)
PAP	0.142	0.134	0.093	Confirmed
BO	0.037	0.034	0.025	Confirmed
AE	0.004	0.001	0.001	Confirmed
OAP	0.046	0.037	0.026	Confirmed

AE: Academic engagement; BO: Burn-out; PAP: Auto-perceived academic performance; OAP: Auto-perceived score.

**Table 6:** Mediation analysis.

	Path Coefficient	*p* Values	Conclusion
DH → AE → PAP	−0.012	0.367	Not supported
DH → AE → OAP	−0.007	0.465	Not supported
DH → BO → PAP	−0.048	0.014	Supported
DH → BO → OAP	−0.027	0.105	Not supported

## Data Availability

The data that support the findings of this study are available on request from the corresponding author.

## Supplementary Materials

The following supporting information can be downloaded at: https://www.mdpi.com/article/10.3390/ijerph22081186/s1, Table S1: Assessment of the measurement model of first-order and second-order constructs (external loadings, validity, and reliability); Table S2: Assessment of the discriminant validity of first-order constructs using Heterotrait-monotrait ratio criterion (HTMT).
