# Supplementary material for: Lots of Digital Files? How Digital Hoarding Is Related to the Academic Performance of University Students
Source: Int J Environ Res Public Health. 2025 Jul 29;22(8):1186. doi: 10.3390/ijerph22081186 (PMC12386574; doi:10.3390/ijerph22081186)
Supplement: Supplementary file 1 [file ijerph-22-01186-s001.zip › ijerph-3761128-supplementary.pdf]

Supplementary File

Table S1. Assessment of the measurement model of first-order and second-order constructs (external loadings, validity, and reliability)

HOC	LOC	Item	Loading	CA	CR	AVE
AE				0.732	0.881	0.787
	AAE			0.627	0.843	0.728
		AAE2	0.862			
		AAE3	0.845			
	VAE			0.767	0.896	0.811
		VAE1	0.892			
		VAE2	0.909			
	DAE			0.821	0.894	0.739
		DAE1	0.901			
		DAE2	0.899			
		DAE3	0.772			
BO				0.818	0.887	0.724
	CBO			0.845	0.907	0.764
		CBO1	0.868			
		CBO2	0.900			
		CBO3	0.854			
	EBO			0.745	0.840	0.569
		EBO1	0.726			
		EBO2	0.801			
		EBO3	0.672			
		EBO4	0.810			
	IBO			1.000	1.000	1.000
		IBO2		1.000	1.000	1.000
DH				0.828	0.895	0.741
	DHA			0.771	0.853	0.593
		DHA1	0.803			
		DHA2	0.811			
		DHA3	0.802			
		DHA4	0.653			
	DHD			0.795	0.859	0.553
		DHD1	0.692			
		DHD2	0.722			
		DHD3	0.844			
		DHD4	0.842			
		DHD5	0.587			
PAP				0.681	0.861	0.756
		R1	0.835			
		R3	0.902			
OAP				1.000	1.000	1.000
		Score	1.000	1.000	1.000	1.000

HOC: Higher order construct; LOC: Lower order construct; CA: Cronbach's Alpha; CR: Composite Reliability; AVE: Average variance extracted.
AE: Academic engagement; BO: Burn-out; DH: Digital hoarding; PAP: Auto-perceived academic performance; OAP: Auto-perceived score.
AAE: Absorption Academic Engagement; VAE: Vigor Academic Engagement; DAE: Dedication Academic Engagement; CBO: Cynicism Academic Burnout; EBO: Exhaustion Academic Burnout; IBO: Inadequacy Academic Burnout; DHA: Digital Hoarding Accumulating; DHD: Digital Hoarding Difficulty deleting.

Table S2. Assessment of the discriminant validity of first-order constructs using Heterotrait-monotrait ratio criterion (HTMT).

	AAE	CBO	DAE	DHA	DHD	EBO	IBO	OAP	PAP
CBO	0.422								
DAE	0.896	0.643							
DHA	0.046	0.077	0.076						
DHD	0.087	0.207	0.139	0.689					
EBO	0.159	0.717	0.392	0.211	0.274				
IBO	0.386	0.794	0.543	0.116	0.188	0.569			
OAP	0.184	0.149	0.183	0.038	0.082	0.174	0.160		
PAP	0.353	0.395	0.438	0.107	0.075	0.374	0.367	0.452	
VAE	0.747	0.470	0.857	0.033	0.087	0.350	0.401	0.118	0.280

AAE: Absorption Academic Engagement; VAE: Vigor Academic Engagement; DAE: Dedication Academic Engagement; CBO: Cynicism Academic Burnout ; EBO: Exhaustion Academic Burnout; IBO: Inadequacy Academic Burnout; DHA: Digital Hoarding Accumulating; DHD: Digital Hoarding Difficulty deleting.
